# Facile and highly precise pH-value estimation using common pH paper based on machine learning techniques and supported mobile devices

**DOI:** 10.1038/s41598-022-27054-5

**Published:** 2022-12-30

**Authors:** Mohamed M. Elsenety, Mahmoud Basseem I. Mohamed, Mohamed E. Sultan, Badr A. Elsayed

**Affiliations:** Department of Chemistry, Faculty of Science, Al-Azhar University, Nasr City, Cairo 11884 Egypt

**Keywords:** Analytical chemistry, Chemistry

## Abstract

Numerous scientific, health care, and industrial applications are showing increasing interest in developing optical pH sensors with low-cost, high precision that cover a wide pH range. Although serious efforts, the development of high accuracy and cost-effectiveness, remains challenging. In this perspective, we present the implementation of the machine learning technique on the common pH paper for precise pH-value estimation. Further, we develop a simple, flexible, and free precise mobile application based on a machine learning algorithm to predict the accurate pH value of a solution using an available commercial pH paper. The common light conditions were studied under different light intensities of 350, 200, and 20 Lux. The models were trained using 2689 experimental values without a special instrument control. The pH range of 1: 14 is covered by an interval of ~ 0.1 pH value. The results show a significant relationship between pH values and both the red color and green color, in contrast to the poor correlation by the blue color. The K Neighbors Regressor model improves linearity and shows a significant coefficient of determination of 0.995 combined with the lowest errors. The free, publicly accessible online and mobile application was developed and enables the highly precise estimation of the pH value as a function of the RGB color code of typical pH paper. Our findings could replace higher expensive pH instruments using handheld pH detection, and an intelligent smartphone system for everyone, even the chef in the kitchen, without the need for additional costly and time-consuming experimental work.

## Introduction

The pH value of different solutions is a particularly important point to determine the optimized conditions and quality control for industrial, biological, chemical, and environmental science either outdoor or indoor applications^1,2^. Hydrogen ions concentration [H^+^] denoted to pH scales from 0 to 14, and the most methods counted for detection are complicated, expensive, and time-consuming as microelectrodes^3^, acid-based indicator^4^, potentiometric titration^1^, colorimetric and fluorescence probes application^5–10^. Currently, potentiometric measurements are the most technique used in pH detection. Where the pH of the solution can be calculated by the measurement of the different voltage between the electrodes of the potentiometric device^11^. Despite, the high accuracy of conventional potentiometric devices, the operation and calibration process is more complicated and costly, which is not applicable to indoor or outdoor purposes. However, the easy and accessible pH strips are used as an alternative method for visual pH detection, but the strips produce lower precise results.

On the other hand, machine learning (ML) techniques give algorithms the ability to predict novel values from training data derived from experiments using Artificial Intelligence (AI). Thus, there are numerous regression or classification algorithms for ML that depend on hyperparameters and mechanisms to achieve their goals and give high performance for planning^12^. ML is being used in chemistry such as chemical discovery^13^, molecular representations^14^, synthetic chemistry^15^, materials chemistry^16^, aquatic chemistry research^17,18^, and water pollution^19^.

Here, the ML technique was used to improve the precision of common strip pH paper. ML models were trained on the 2689 experimental data which covered the whole pH range. Further, we developed a mobile/web application based on ML algorithms to predict the pH values. Therefore, the developed app could work on mobile which could be used as portable devices for anyone (whether a chemist or not) without additional costs, fast response, and is applicable for different applications.

## Materials and apparatus

Acetic acid, phosphoric acid, boric acid, HCl, and NaOH were used without any further purification (Sigma Aldrich). Universal indicator pH paper (1–14) Q/3211821AB001-2002 (China). The pH measurements were carried out on a 3520 pH Meter (JENWAY, England). Mastech MS6612 Digital Luxmeter Illuminometer Light (Range Peak 200,000 Lux) was used for measuring the light intensity in the experimental workplace.

### pH buffer solution

Universal Britton–Robinson (B–R) buffer was prepared as reported^20^. Briefly, the stock aqueous B-R buffer solutions (pH = 2.86) by mixing equal molar ratio (1:1:1) of 0.02 mol/L from acetic acid, phosphoric acid, and boric acid. Dropwise of 0.20 mol/L of NaOH or 0.20 mol/L of HCl was used for adjusting the pH values (interval = 0.10) to cover the whole pH range.

### Machine learning algorithms

Regression is a technique used for prediction continues pH values learning and figuring out causal relations between the actual and prediction pH values. Eleven supervised machine learning regression models were applied to the data collected and choose the best model that fits with the selected problem, including Linear Regression (LR), Decision Tree Regressor (DT_*R*_), Random Forest Regressor (RT_*R*_), K Neighbors Regressor (KNN_*R*_), Support Vector Regression (SVR), Lasso regression (L_1_), Ridge Regression (L_2_), Elastic Net regressor (EN_*R*_), AdaBoost Regression (AB_*R*_), Gradient Boosting Regressor (GB_*R*_), and Artificial Neural Network Regressor (ANN_*R*_). All models can be found in Scikit-learn in the class model^21^. In addition, the data visualization of exploratory data analysis and heatmap figures were created using the seaborn package based on python code^22^.

### Metrics for regression

Several metrics were used for evaluating the regression models, coefficient of determination (R^2^), Mean Squared Error (MSE), Mean Absolute Error (MAE), and Root Mean Square Error (RMSE) can be calculated Scikit-learn in class metrics according to Eqs. (1–4).^23,24^

1 $${R}^{2}=1-\frac{{\sum }_{i} {\left({y}_{i}-{\hat{y}}_{i}\right)}^{2}}{{\sum }_{i} {\left({y}_{i}-{\overline{y}}\right)}^{2}}$$

2 $$MSE=\frac{1}{N}{\sum }_{i=1}^{N} {\left({y}_{i}-{\hat{y}}_{i}\right)}^{2}$$

3 $$MAE=\frac{1}{N}{\sum }_{i=1}^{N} \left|{y}_{i}-{\hat{y}}_{i}\right|$$

4 $$RMSE=\sqrt{\frac{{\sum }_{i=1}^{N} {\left({y}_{i}-{\hat{y}}_{i}\right)}^{2}}{N}}$$

where *N* is the number of recorded samples, *y*_*i*_ is the predicted pH value, and $\hat{y}_{i}$ is the actual pH value.

### Automated color-information-extraction from the captured images

**Figure 1:**
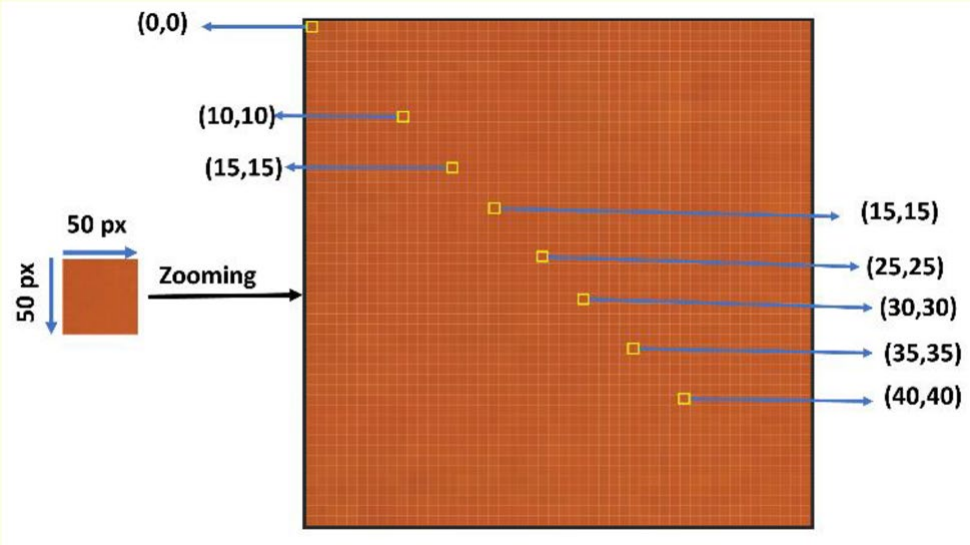
Pixel positions of pH paper image (at pH value = 4 , as an example).

To extract the color code (RGB) from images, we used a Python 3.7 code based on the OpenCV package to extract the RGB for each image^25^. We noted a small deviation of the RGB values at various positions in one image. Thus, the RGB values were estimated at seven distinct (X, Y) positions (10,10;15,15; 20,20; 25,25; 30,30; 35,35; 40,40) to cover the whole image as illustrated in Fig. 1.

### pH discrimination with a machine learning model

We exploited a KNN regression model-based machine learning algorithm to study 2689 collected sample data using Python 3.7 and the scikit-learn package^26,27^. We randomly separated the data into training data (70%, i.e., 1880 samples) and testing data (30%, i.e., 808 samples). In the inference model training phase, the testing data was completely excluded. Furthermore, in machine learning, hyperparameters are those parameters that are explicitly provided by the user to influence the learning process and improve the learning of the model. Thus, we trained our models using a series of integer number [1,2,3,….], and as a result of that the optimal hyperparameters (highest coefficient, and lower errors) was found when we used K = 5.

## Result and discussion

**Figure 2:**
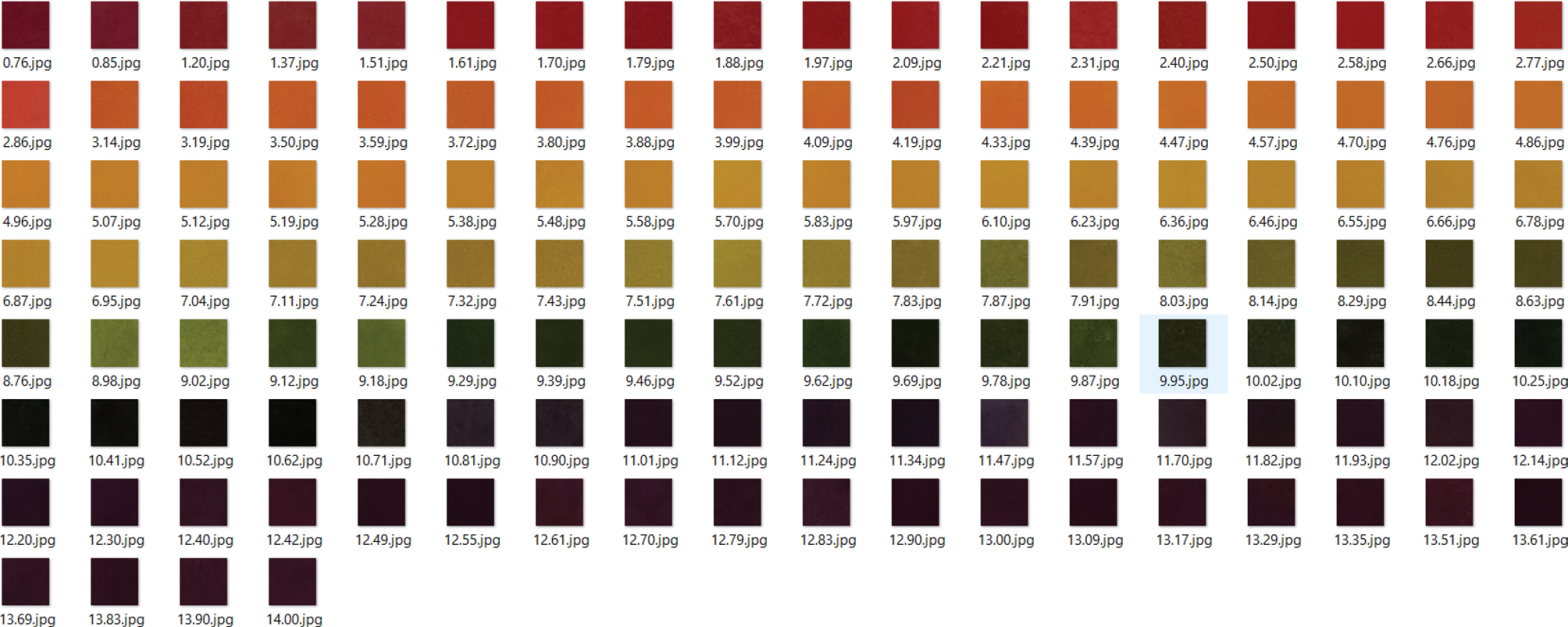
Samples of the captures of an experimentally colored change of the pH-paper at 350 Lux.

Figure 2 presents collections of 130 captures of an experimentally colored change of the pH paper (at 350 Lux) in the range of (0–14) by an interval of ~ 0.1 pH-value. It is worth mentioning that the traditional estimation based on the color change of pH paper is accompanied by a significant variance in pH value (~ 2). This high variance of pH value led to a noteworthy wrong estimation by eye detection. This finding encourages us to develop a new simple and more precise method for pH-value detection. Thus, the experiments were extended to cover most of the three different illumination workplaces at 350, 200, and 20 Lux, that the user could work on. Moreover, the homogeneity of the color of the pH paper was emphasized by the collected color RGB code for seven distinct positions per capture. In total, the data set includes 2689 experimental RGB values from different illumination workplaces.

**Figure 3:**
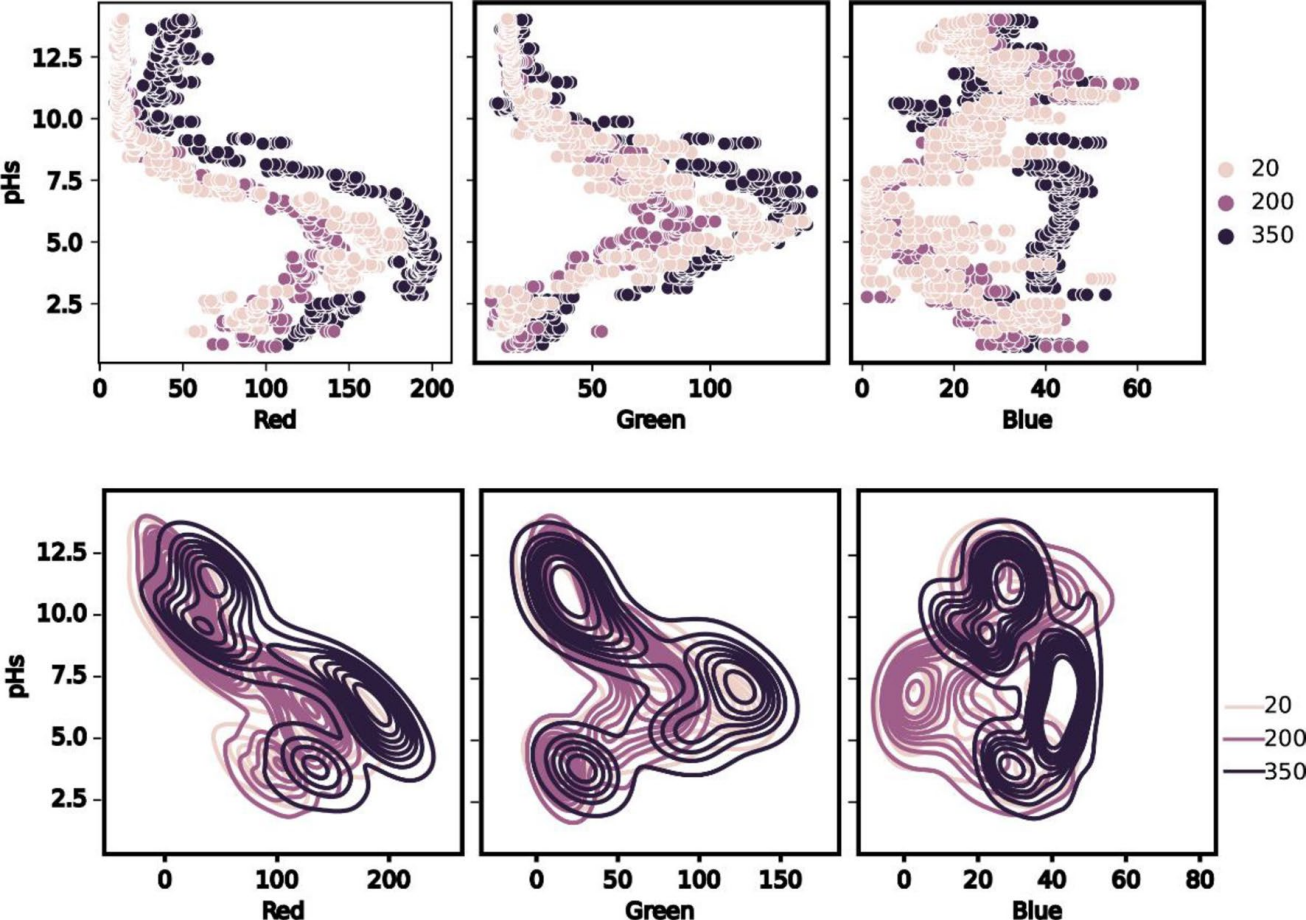
Exploratory Data Analysis of changed RGB code in different illuminated workplaces at (20, 200, and 350 Lux).

To better understand the observed results in the different workplaces, Exploratory Data Analysis (EDA) of color code RGB against pH values with respect to different light intensities at 20, 200, and 350 Lux, was illustrated in Fig. 3.

The color code points were collected in three parts in a wide pH range. The significant changes in the color code of Red and Green or even Blue were in the range of (2.5: 9) pH values at the three different investigated workplaces of light intensities at (20, 200, and 350 Lux). It is worth mentioning, that the blue color code at low-intensity light of 20 Lux (a little dark workplace) deviates from those obtained in higher or medium light intensity, which suggests avoiding future testing in low light conditions. In contrast, the results revealed no significant difference between the behavior of Red or Green colors at light intensity. The results show the increase in basicity (> 9) or increase in acidity and (< 2.5) could interpret the color and may produce less accurate prediction in that part of the pH range. Thus, this finding may encourage the scientific community to prepare higher sensitive material to work in strong acid and/or Strong base medium.

Furthermore, it is critical to recognize and evaluate how dependent each parameter is on the others. This knowledge can aid in the definition of the expectations that these interdependencies provide, leading to the creation of more effective pH devices and color-sensitive materials. Because of this, using a machine learning strategy, the statistical Pearson’s correlation coefficients (r_x,y_) between the pH parameters were investigated based on the following Eqs. (5) and (6):

5 $${\mathrm{cov}}_{x,y}=\frac{\sum \left({x}_{i}-{\overline{x}}\right)\left({y}_{i}-{\overline{y}}\right)}{N-1}$$

6 $${r}_{xy}=\frac{{\sum }_{i=1}^{N} \left({x}_{i}-{\overline{x}}\right)\left({y}_{i}-{\overline{y}}\right)}{\sqrt{{\sum }_{i=1}^{N} {\left({x}_{i}-{\overline{x}}\right)}^{2}}\sqrt{{\sum }_{i=1}^{N} {\left({y}_{i}-{\overline{y}}\right)}^{2}}}$$

where *N* number of recorded samples, ${x}_{i}$, ${y}_{i}$ are individual elements of RGB and pH predicted values respectively, and $\overline{y}$ the mean value of pH values.

**Figure 4:**
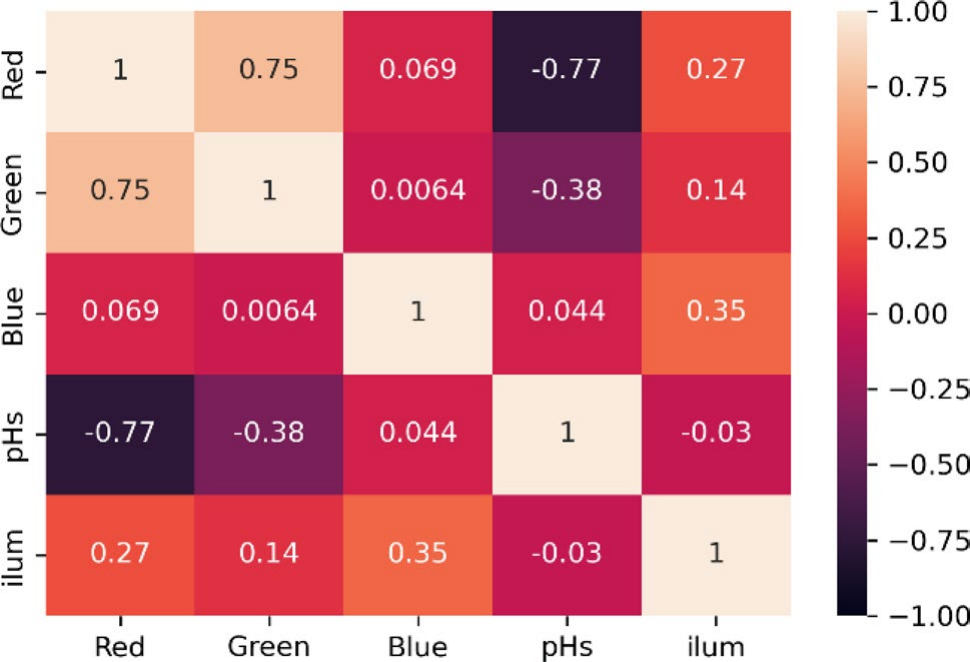
Pearson’s correlation coefficients between the pH parameters.

The correlation between the pH parameters was presented with a heatmap in Fig. 4. The obtained results reflect an excellent higher negative correlation between the pH values with Red color (−0.77). In the same way, an acceptable correlation of pH value with the green color by (−0.38). The blue color showed an incredibly low correlation with pH value (0.044) from those observed in the red or green colors. This refers to that the blue color will have a small effect on the machine learning prediction compared to the red and green colors. In the same way, the illumination of workplaces has no significant effect on the pH value by −0.03. Thus, the colored pH paper can be safely captured whatever the light intensity.

### ML model prediction

**Figure 5:**
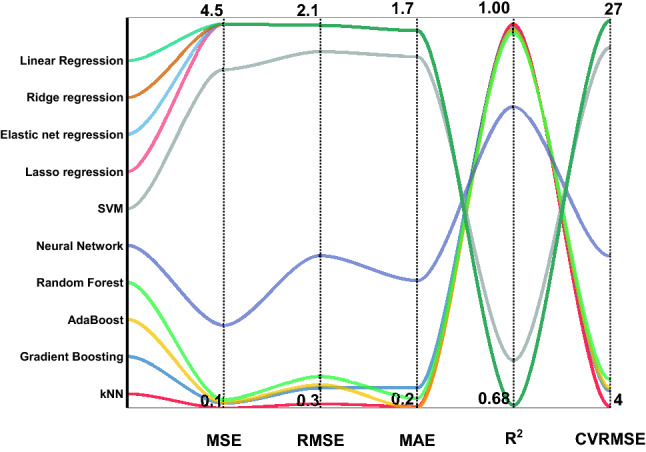
Output results of performed regression algorithms of Linear, Ridge, Lasso, Elastic Net, Polynomial, Support vector machine (SVM) Regresso, Gradient Boosting, AdaBoost, and Random Forest Regressor.

Using experimental data, a preliminary analysis of machine learning regression techniques was performed with optimal hyperparameters on K-Nearest Neighbors (KNN), Linear, Lasso, Elastic Net, AdaBoost, Neural Network, Random Forest, and Support vector machine (SVM), and Gradient Boosting Regressor algorithms^28–30^ to estimate coefficients of determination (R^2^) and the minimum errors of the corresponding regression evaluation metrics concerning root mean squared error (RMSE), mean absolute error (MAE), and mean squared error (MSE) as shown in Fig. 5 and recorded in Table 1.

**Table 1:** Regression evaluation metrics of performed algorithms on the experimental data (cross-validation with K-fold = 5).

	MSE	RMSE	MAE	R^2^	CVRMSE
kNN	0.071	0.266	0.160	0.995	3.385
AdaBoost	0.116	0.341	0.157	0.992	4.332
Gradient Boosting	0.130	0.361	0.242	0.991	4.590
Random Forest	0.164	0.405	0.196	0.988	5.143
Neural Network	0.794	0.891	0.578	0.943	11.321
SVM	3.584	1.893	1.460	0.744	24.058
Ridge regression	4.399	2.097	1.637	0.686	26.651
Linear Regression	4.399	2.097	1.637	0.686	26.651
Elastic net regression	4.399	2.097	1.637	0.686	26.651

It's obvious that the KNN model with optimal hyperparameters of five points performs significant result of R^2^ (0.993) combined with the lowest errors of MSE, RMSE, and MAE (0.012, 0.320, and 0.182, respectively) compared to other models. In addition, the coefficient of the variation of the root means square error (CVRMSE) of KNN models shows a higher stability performance of 4.077 compared to other models. Further, the cross-validation with K-fold of (3, 5, 10, and 20) was tested for confirming the stability of the models. However, no significant difference was found between the results, which verified the KNN models.

**Figure 6:**
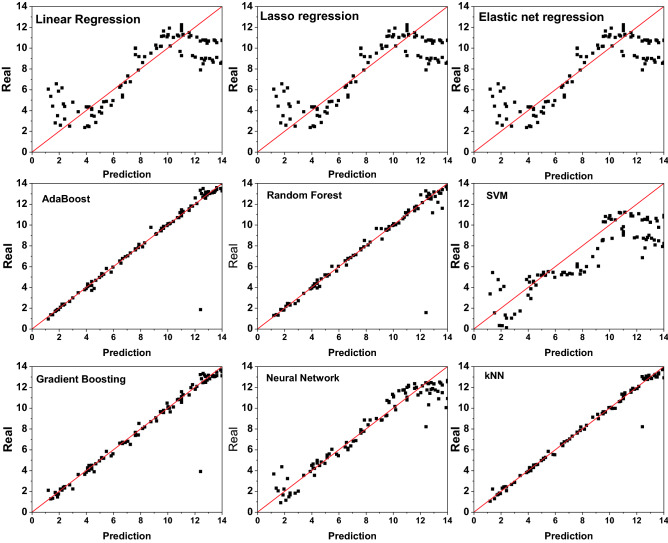
The model's prediction results (based on the test data) vs. experimental results.

To deepen understanding, further investigation showed that the results of the model's prediction (based on test data) vs the experimentally obtained pH values are represented in the scatter plot in Fig. 6. The linear regression, elastic net, and Neural network algorithms could not recognize the whole experimental points, especially at the strong acid/base pH range. However, a precise estimate would be placed along a square-diameter line using KNN, Gradient boosting, Random Forest, and AdaBoost algorithms, which could be selected for further steps of deploying the code. Despite the higher performance and exceedingly small deviation of those algorithms, the KNN was chosen for deploying the machine learning mobile application due to having the lowest errors (RMSE; 0.32) and higher stability (CVRMSE; 4.08) as well.

It is now clear that the KNN model can successfully show the underlying patterns of the color RGB code in the pH value estimation based on experimental data collections. Thus, the machine learning approach based on this model was further expanded and used to develop a versatile platform able to predict the pH value using common pH paper with high accuracy. The online mobile application of the prediction model was developed using python code and streamlit cloud (freely available) and permits the highly predicted determination of the pH value as a function of the RGB color code of common pH paper.

**Figure 7:**
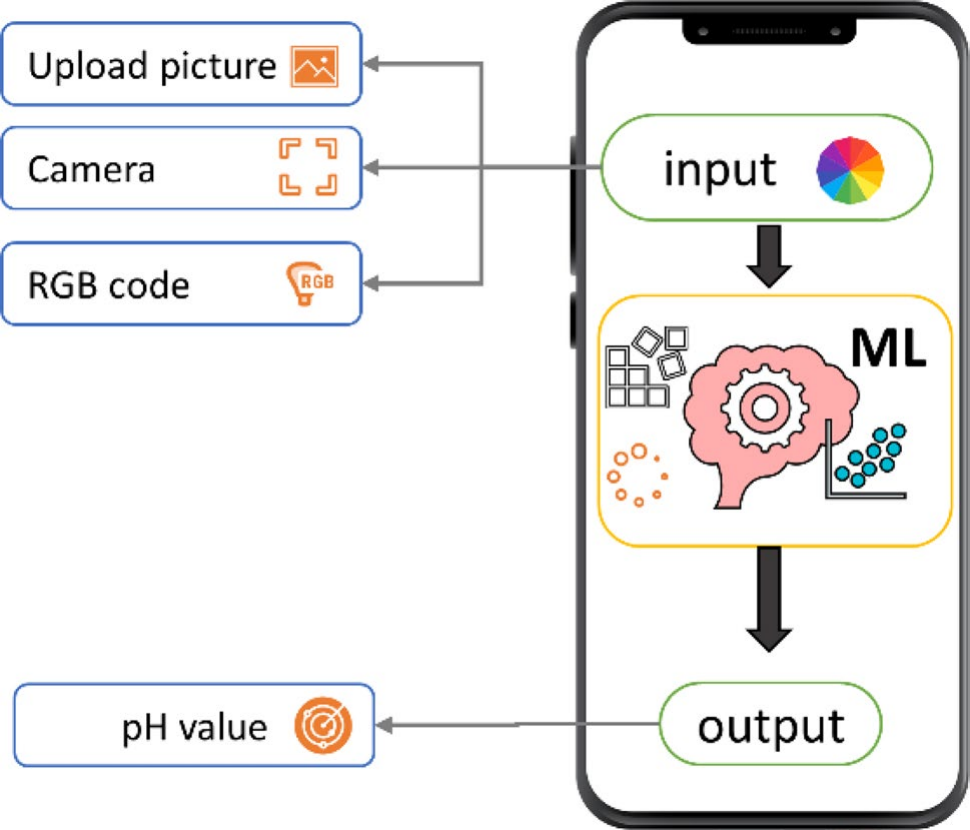
Schematic process of pH detection using ML.

As illustrated in Fig. 7 the mobile application includes three steps; starting with the input file which could be able to insert the pH paper capture (after being immersed in the target solution immediately). For more facility, we have coded three options (upload a picture, use a mobile camera, or insert a RGB color code). This step is followed by a built-in Machin learning process (without control from the user). Finally, the output of the pH value will appear on the screen.

**Figure 8:**
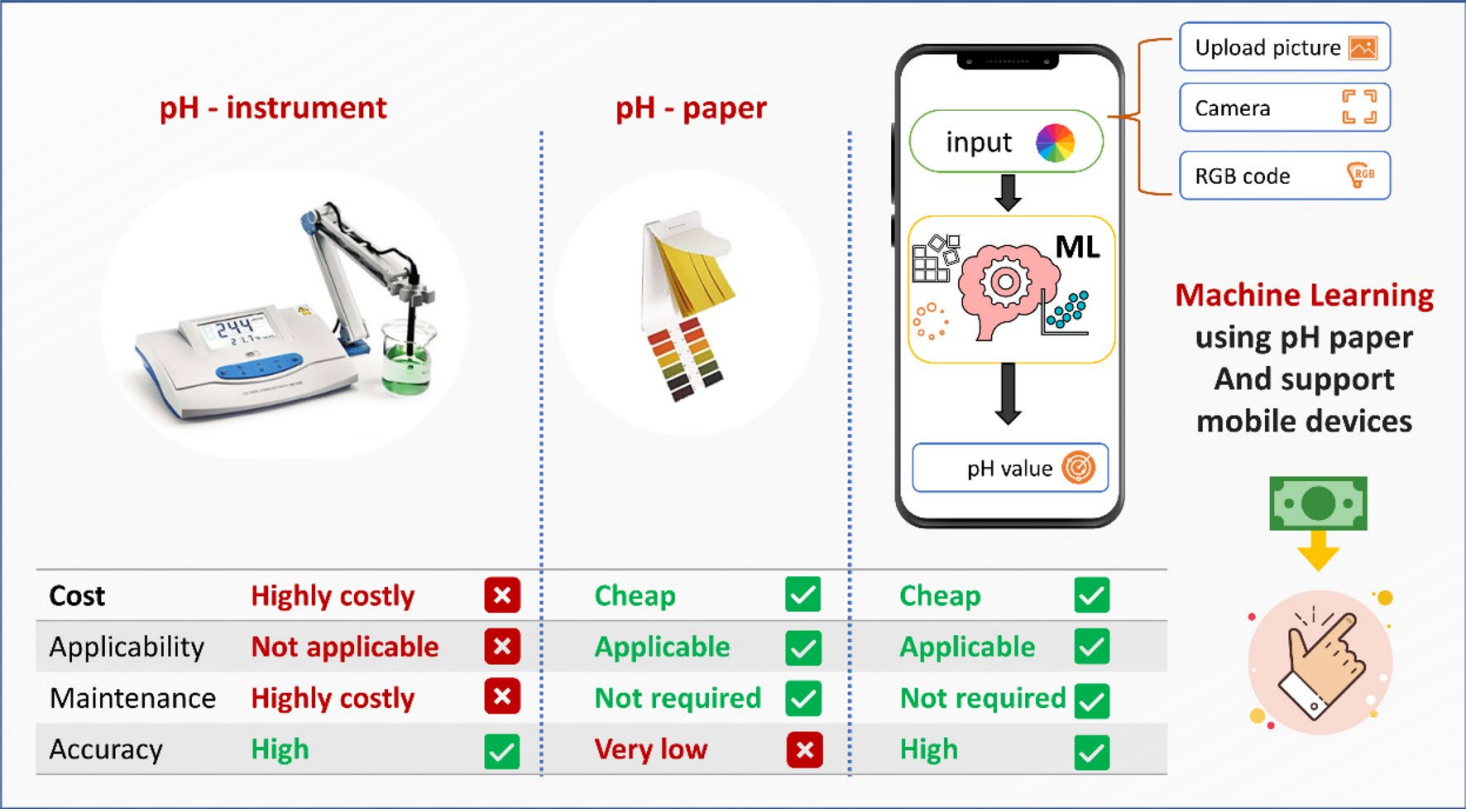
Comparison of pH instrument, pH paper, and the current study.

Our study has a significant advantage over what is already used, Fig. 8 shows the fair comparison of pH instruments, pH paper, and the current study.

**Figure 9:**
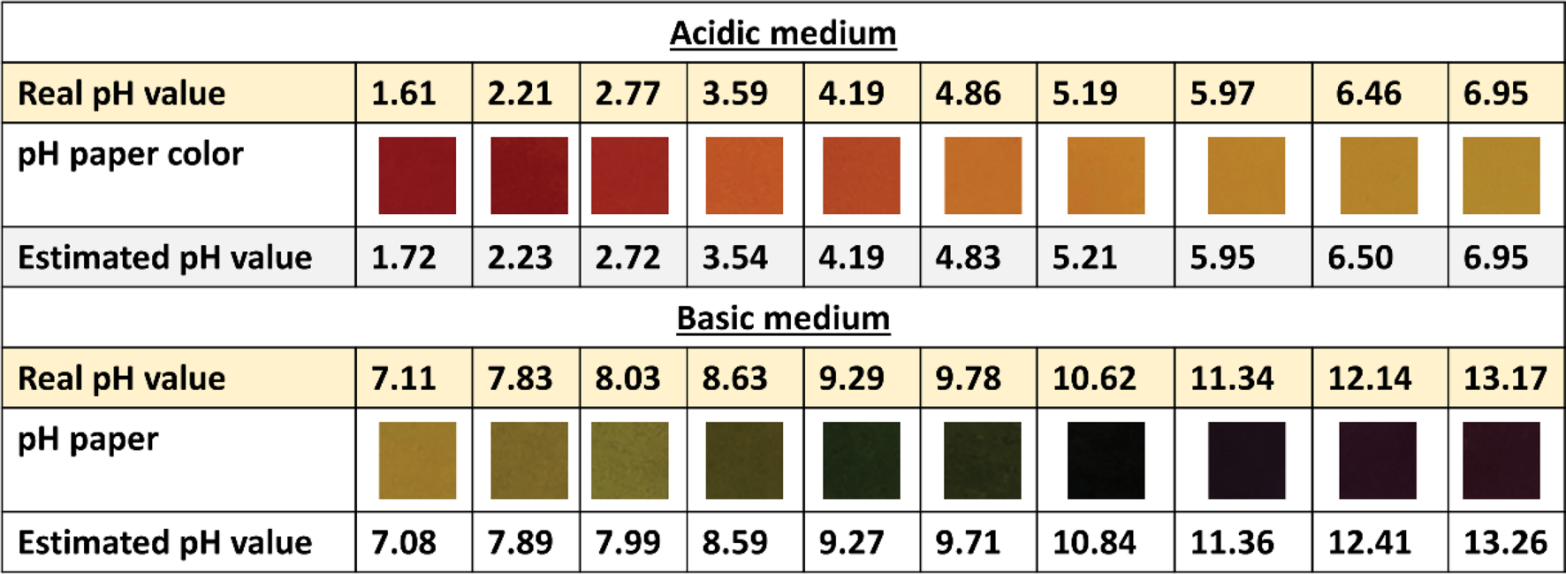
Estimated pH value from mobile application in comparison with real one.

Furthermore, Fig. 9 shows the estimated pH value (output results) of the proposed mobile application in comparison with the real pH value. Interestingly, this correlation between real and estimated values in the whole range of pH (acid or base) is related to the higher accuracy of the used ML model.

However, Solmaz et al.^31^ studied pH strips colorimetric detection using ML, as presented in Table 2.

**Table 2:** The advantages of the current work that related studies.

	Solmaz et al. work^31^	Current work
Type of pH	pH strips	Universal pH paper
Cost	A little higher	Very cheap
Type of ML techniques	Classification	Regression
Input data	450	2688
Illumination source	Special tools required	In ambient condition
Illumination effect	Not studied	Studied at 350, 200, 20 Lux of light intensities
pH range studied	4: 9	Whole range 0: 14
Accuracy	Low (estimated pH value ± 1)	Very high (estimated pH value ± 0.1)

**Figure 10:**
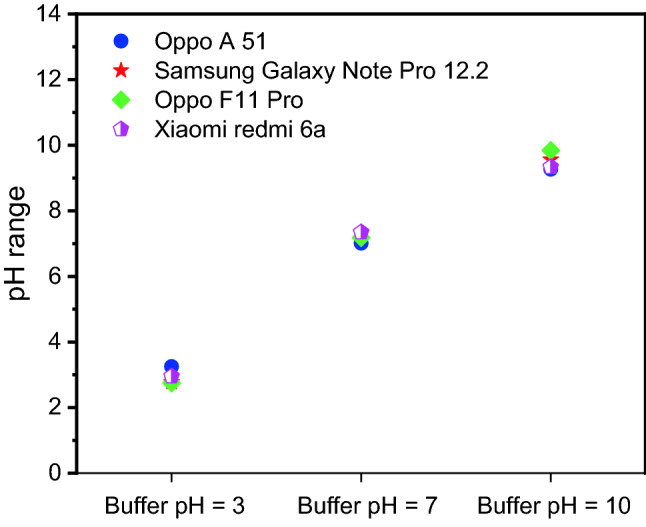
Estimated pH value from different smartphones.

However, four different types of smartphones were used to check the accuracy of pH value predictions for three buffer solutions (pH = 3, 7, and 10). The default setting was used to avoid any smartphone effects. As shown in Fig. 10 and Table 3, the various smartphones do have no significantly different pH value estimations with an accuracy of more than 90% for each type.

**Table 3:** Accuracy and estimated pH value from different smartphones.

Xiaomi redmi 6a	Oppo F11 Pro	Samsung Galaxy Note Pro 12.2	Oppo A 51	Expected value	
2.951	2.75	2.77	3.25	3	
**98%**	**91%**	**92%**	**92%**		**Accuracy**
7.3461	7.1845	7.32	7.013	7	
**95%**	**97%**	**96%**	**100%**		**Accuracy**
9.3348	9.84	9.56	9.27	10	
**93%**	**98%**	**95%**	**92%**		**Accuracy**

Significant values are in bold.

Furthermore, Table 4 shows recommended conditions and limitations for using the application to achieve more accurate predictions.

**Table 4:** Recommended mesurment conditions for application users.

Light intensity workplace	Normal brightness (200:400 Lux)
pH -paper	Universal indicator pH paper (1–14) Q/3211821AB001-2002
Distance between smartphone and pH paper	15:20 cm
Smartphone camera resolution	More than 5 MP
Smartphone camera operation	Default capture without effects or using different software
Smartphone capture centering	The pH paper (under test) should be at the center position of the smartphone camera

Overall, the present findings solve the problem of pH accuracy using common pH paper without the need for additional costly and time-consuming experimental work. However, our approach solves the problems of excessive cost and maintenance required for traditional pH meters.

## Conclusion

The findings demonstrate a strong negative association between pH values and both the red color (−0.77) and the green color (−0.38). The blue color will have an insignificant impact on machine learning prediction which revealed a low correlation (0.044). The KNN model exhibits significant R^2^ (0.993) results along with the lowest MSE, RMSE, and MAE errors (0.012, 0.320, and 0.182, respectively). This paper also demonstrated the potential of the ML approach to estimate the pH value of solutions using common pH paper. We developed a freely available application that supported mobile devices to predict the pH value based on ML and using common pH paper with precise results. Future research should consider the preparation of new optical material with extremely sensitive color changes in a strong acid/base medium.

## Data Availability

The web application and mobile application are freely available “https://elsenety-ph4-ph-app4-pazg10.streamlitapp.com” or “https://soft.yallascience.com/2018/06/researcher-tools-software.html”. The ML code and RGB data are available on request due to privacy/ethical restrictions from the corresponding author [Mohamed M. Elsenety; m.elsenety@azhar.edu.eg].

## References

[1] Wilson GS (2002). Measurement of pH. Definition, standards, and procedures. Pure Appl. Chem.

[2] Jensen WB, Ault B (2004). The symbol for pH. J. Chem. Educ..

[3] Cha CS, Li CM, Yang HX, Liu PF (1994). Powder microelectrodes. J. Electroanal. Chem..

[4] Zoromba MS (2017). Novel and economic acid-base indicator based on (p-toluidine) oligomer: Synthesis; characterization and solvatochromism applications. Spectrochim. Acta Part A Mol. Biomol. Spectrosc..

[5] Elmorsi TM, Aysha TS, Machalický O, Mohamed MBI, Bedair AH (2017). A dual functional colorimetric and fluorescence chemosensor based on benzo[f]fluorescein dye derivatives for copper ions and pH; kinetics and thermodynamic study. Sens. Actuators B Chem..

[6] Mohamed MBI (2020). Colorimetric chemosensor and turn on fluorescence probe for pH monitoring based on xanthene dye derivatives and its bioimaging of living *Escherichia coli* bacteria. J. Fluoresc..

[7] Aysha TS, El-Sedik MS, Mohamed MBI, Gaballah ST, Kamel MM (2019). Dual functional colorimetric and turn-off fluorescence probe based on pyrrolinone ester hydrazone dye derivative for Cu^2+^ monitoring and pH change. Dye. Pigment..

[8] Aysha TS, Mohamed MBI, El-Sedik MS, Youssef YA (2021). Multi-functional colorimetric chemosensor for naked eye recognition of C^u2+^, Zn^2+^ and Co^2+^ using new hybrid azo-pyrazole/pyrrolinone ester hydrazone dye. Dye. Pigment..

[9] Elsayed BA, Ibrahem IA, Attia MS, Shaaban SM, Elsenety MM (2016). Highly sensitive spectrofluorimetric analysis and Molecular Docking using benzocoumarin hydrazide derivative doped in sol-gel matrix as optical sensor. Sens. Actuators B Chem..

[10] Elsenety MM, Elsayed BA, Ibrahem IA, Bedair MA (2020). Photophysical, DFT and molecular docking studies of Sm(III) and Eu(III) complexes of newly synthesized coumarin ligand. Inorg. Chem. Commun..

[11] Kim H (2021). Fluorescent sensor array for high-precision pH classification with machine learning-supported mobile devices. Dye. Pigment..

[12] Mekonnen Y, Namuduri S, Burton L, Sarwat A, Bhansali S (2020). Review—machine learning techniques in wireless sensor network based precision agriculture. J. Electrochem. Soc..

[13] Qu X, Latino DARS, Aires-De-sousa J (2013). A big data approach to the ultra-fast prediction of DFT-calculated bond energies. J. Cheminform..

[14] Raghunathan S, Priyakumar UD (2022). Molecular representations for machine learning applications in chemistry. Int. J. Quantum Chem..

[15] Pflüger PM, Glorius F (2020). Molecular machine learning: The future of synthetic chemistry?. Angew. Chemie Int. Ed..

[16] Raccuglia P (2016). Machine-learning-assisted materials discovery using failed experiments. Nature.

[17] He L (2021). Applications of computational chemistry, artificial intelligence, and machine learning in aquatic chemistry research. Chem. Eng. J..

[18] Li L, Rong S, Wang R, Yu S (2021). Recent advances in artificial intelligence and machine learning for nonlinear relationship analysis and process control in drinking water treatment: A review. Chem. Eng. J..

[19] Chen H (2020). Kernel functions embedded in support vector machine learning models for rapid water pollution assessment via near-infrared spectroscopy. Sci. Total Environ..

[20] Britton HTS, Robinson RA (1931). Universal buffer solutions and the dissociation constant of veronal. J. Chem. Soc..

[21] Pedregosa F (2011). Scikit-learn: Machine learning in Python. J. Mach. Learn. Res..

[22] seaborn. Available at: https://seaborn.pydata.org/generated/seaborn.heatmap.html.

[23] Awan MJ (2021). Cricket match analytics using the big data approach. Electronics.

[24] Zakeri-Nasrabadi M, Parsa S (2022). Learning to predict test effectiveness. Int. J. Intell. Syst..

[25] Yu Q, Cheng HH, Cheng WW, Zhou X (2004). Ch OpenCV for interactive open architecture computer vision. Adv. Eng. Softw..

[26] Guo G, Wang H, Bell D, Bi Y, Greer K (2005). Using kNN model for automatic text categorization. Soft Comput..

[27] Pedregosa FABIANPEDREGOSA, F (2011). Scikit-learn Scikit-learn: Machine learning in Python. J. Mach. Learn. Res..

[28] Tao Q, Xu P, Li M, Lu W (2021). Machine learning for perovskite materials design and discovery. NPJ Comput. Mater..

[29] Yılmaz B, Yıldırım R (2021). Critical review of machine learning applications in perovskite solar research. Nano Energy.

[30] She C (2021). Machine learning-guided search for high-efficiency perovskite solar cells with doped electron transport layers. J. Mater. Chem. A.

[31] Mutlu AY (2017). Smartphone-based colorimetric detection: Via machine learning. Analyst.

